# Supplementary material for: A review of the effectiveness of operational curtailment for reducing bat fatalities at terrestrial wind farms in North America
Source: PLoS One. 2021 Nov 17;16(11):e0256382. doi: 10.1371/journal.pone.0256382 (PMC8598023; doi:10.1371/journal.pone.0256382)
Supplement: S1 Appendix — (DOCX) [file pone.0256382.s003.docx]

**S2 Appendix: R Code for Analysis**

Meta-analysis Modeling and Meta-analysis Power Analysis R Code
#Libraries
library(dplyr)
library(tidyr)
library(broom)
library(ggplot2)
library(purrr)
library(doBy)
library(meta)
library(multcomp)
library(reshape2)
library(metafor)
library(ggpubr)
library(mice)
library(qpcR)
library(foreach)
library(MuMIn)

#Data management
#import data – this has already been converted to experiment-control pairings (Table 1). In instances where a particular study tested multiple experimental cut-in speeds, control data was duplicated such that each experimental row had a corresponding control row of data.
#pull .csv file included with the report into your working database then add to your R environment
data <- read.csv("data/UsableData_09242019.csv")
#remove data that doesn't have a "Study" - which in reality is just the Wild Cat multiple years of #experimental (averaged prior to import in excel)
data <- data %>% drop_na(Study)
#format Study and Curtailment ID as factor (categorical variables)
data$Study <- as.factor(data$Study)
data$CurtID <- as.factor(data$CurtID)

#project information – this is the study site level information, import it as before
studies <- read.csv("data/Studies_ecoregion.csv")
studies$CurtID <- as.factor(studies$CurtID)
#join study info to data based on the unique curtailment experiment number
data <- merge(data, studies, by="CurtID", all.x = T)

#select only the columns we need
data <- data[, c("CurtID", "TreatmentID", "Study", "Project.x", "Year", "Lat", "Long", "Treatment.Type", "ControlType", "Hr.night", "Search.Int", "cut.in", "n.turbines", "Feathering", "Start", "End", "Nights", "num.found", "bats.turbine", "bats.turbine.lci", "bats.turbine.uci", "estimator", "conf.level", "pct.decrease", "pct.decrease.lci", "pct.decrease.uci", "MW", "NumTurb", "HH", "RD", "NA_L1NAME", "NA_L2NAME")]

##data setup – change controls and treatments from separate rows to columns of one row
controls <- subset(data, Treatment.Type == "control")
experiments <- subset(data, Treatment.Type == "experimental")
#information we need to pull from controls and add: cut.in, Year, start, end, nights, num found, bats.turbine, bats.turbine.lci, bats.turbine.uci, pct.decrease, pct.decrease.lci, pct.decrease.uci
controls <- controls[, c("Study", "cut.in", "Year", "Start", "End", "Nights", "num.found", "bats.turbine", "bats.turbine.lci", "bats.turbine.uci", "pct.decrease", "pct.decrease.lci", "pct.decrease.uci")]
#rename columns in the control data to distinguish from treatments
names(controls)[2] <- "control.cutin"
names(controls)[3] <- "Year.cont"
names(controls)[4] <- "Start.cont"
names(controls)[5] <- "End.cont"
names(controls)[6] <- "Nights.cont"
names(controls)[7] <- "num.found.cont"
names(controls)[8] <- "cont.bats.turbine"
names(controls)[9] <- "cont.bats.turbine.lci"
names(controls)[10] <- "cont.bats.turbine.uci"
names(controls)[11] <- "cont.pct.decrease"
names(controls)[12] <- "cont.pct.decrease.lci"
names(controls)[13] <- "cont.pct.decrease.uci"
#merge based on "Study" so that treatment and control information are separate columns
data.upd <- merge(experiments, controls, by = "Study", all=TRUE)

#set up additional columns and calculate delta cutin (different between treatment and control cut-in)
data.upd$delta.cutin <- data.upd$cut.in - data.upd$control.cutin
#turning estimates from bats per turbine into bats per turbine hour (because a few studies implemented curtailment only for part of the night)
#treatment mean
data.upd$exp.bats.th <- data.upd$bats.turbine/ (data.upd$Nights * data.upd$Hr.night)
#treatment lower CI
data.upd$exp.bats.th.lci <- data.upd$bats.turbine.lci/ (data.upd$Nights * data.upd$Hr.night)
#treatment upper CI
data.upd$exp.bats.th.uci <- data.upd$bats.turbine.uci/ (data.upd$Nights * data.upd$Hr.night)
#control mean
data.upd$cont.bats.th <- data.upd$cont.bats.turbine/ (data.upd$Nights * data.upd$Hr.night)
#control lower CI
data.upd$cont.bats.th.lci <- data.upd$cont.bats.turbine.lci/ (data.upd$Nights * data.upd$Hr.night)
#control upper CI
data.upd$cont.bats.th.uci <- data.upd$cont.bats.turbine.uci/ (data.upd$Nights * data.upd$Hr.night)

#Setting up Delta cut-in categories
#create a new column
data.upd$delta.cutin.cat <- NA
#if delta cut-in is < 1.4 m/s, categorized as "A" or "Category 1"
data.upd$delta.cutin.cat[data.upd$delta.cutin < 1.4] <- "A"
#if delta cut-in is > 1.4 m/s but < 2.6, categorized as "B" or "Category 2"
data.upd$delta.cutin.cat[data.upd$delta.cutin > 1.4 & data.upd$delta.cutin < 2.6] <- "B"
#if delta cut-in is >2.5 1.4 m/s, categorized as "C" or "Category 3"
data.upd$delta.cutin.cat[data.upd$delta.cutin > 2.5] <- "C"
#change this to a categorical variable
data.upd$delta.cutin.cat <- as.factor(data.upd$delta.cutin.cat)

#Calculate Fatality Ratio and Associated SE for each study
#subset to only include data with estimate of bats.turbine
data.ratio <- subset(data.upd, bats.turbine > 0)
data.percent <- subset(data.upd, is.na(bats.turbine))
#now lets calculate the fatality ratio
data.ratio$mort.ratio <- data.ratio$exp.bats.th / data.ratio$cont.bats.th
data.percent$mort.ratio <- 1 - data.percent$pct.decrease

#calculate standard error for data with confidence interval estimates
#control
#create column for control standard error
data.ratio$cont.se <- NA
#for values with a 95% confidence interval, convert CI to SE
data.ratio$cont.se[data.ratio$conf.level == 0.95] <- (data.ratio$cont.bats.th.uci[data.ratio$conf.level == 0.95]-data.ratio$cont.bats.th.lci[data.ratio$conf.level == 0.95])/3.92
#for values with a 90% confidence interal, convert CI to SE
data.ratio$cont.se[data.ratio$conf.level == 0.90] <- (data.ratio$cont.bats.th.uci[data.ratio$conf.level == 0.90]-data.ratio$cont.bats.th.lci[data.ratio$conf.level == 0.90])/3.29

#experimental
#create column for treatment standrad error
data.ratio$exp.se <- NA
#for values with a 95% confidence interval, convert CI to SE
data.ratio$exp.se[data.ratio$conf.level == 0.95] <- (data.ratio$exp.bats.th.uci[data.ratio$conf.level == 0.95]-data.ratio$exp.bats.th.lci[data.ratio$conf.level == 0.95])/3.92
#for values with a 90% confidence interval, convert CI to Se
data.ratio$exp.se[data.ratio$conf.level == 0.90] <- (data.ratio$exp.bats.th.uci[data.ratio$conf.level == 0.90]-data.ratio$exp.bats.th.lci[data.ratio$conf.level == 0.90])/3.29

#create a column for combined standard error (treatment and control)
data.ratio$se.combo <- NA

if(data.ratio$conf.level > 0) {
 data.ratio$se.combo <- abs(data.ratio$mort.ratio)*(sqrt((data.ratio$exp.se/data.ratio$exp.bats.th)^2 + (data.ratio$cont.se/data.ratio$cont.bats.th)^2))}
data.ratio$rr.se <- NA

if(data.ratio$conf.level > 0) {
 data.ratio$rr.se <- sqrt((data.ratio$exp.se)^2/(data.ratio$exp.bats.th)^2 + (data.ratio$cont.se)^2/(data.ratio$cont.bats.th)^2)}

#lets create the same variables for the percent only data
data.percent$cont.se <- NA
data.percent$exp.se <- NA
data.percent$se.combo <- NA
data.percent$mort.ratio.uci <- NA
data.percent$mort.ratio.lci <- NA
data.percent$rr.se <- NA
#merge data back together
data.ratio <- rbind(data.ratio, data.percent)

####Scale covariates
#scale control cut-in speed
data.ratio $scale.control.cutin <- as.vector(scale(data.ratio $control.cutin))

#scale delta cutin
data.ratio $scale.delta.cutin <- as.vector(scale(data.ratio $delta.cutin))

#scale rotor diameter
data.ratio $scale.rd <- as.vector(scale(data.ratio $RD.x))

#Add ecoregion as a covariate because of the number in each category, Level 2 ecoregions need to be #grouped based on general geographic region
#Group 1 (Northeast) = Atlantic Highlands + Mixed Wood Plains; Group 2 (East)= Ozark/Ouachita-Appalachian forest; Group 3 (West/Midwest) = Central plains, temperate praries, west-central semiarid praries
data.ratio $ecoregion <- NA
data.ratio $ecoregion <- "Midwest/West"
data.ratio $ecoregion[data.ratio $NA_L2NAME.x == "ATLANTIC HIGHLANDS" | data.ratio $NA_L2NAME.x == "MIXED WOOD PLAINS"] <- "Northeast"
data.ratio $ecoregion[data.ratio $NA_L2NAME.x == "OZARK/OUACHITA-APPALACHIAN FORESTS"] <- "MidEast"
#turn this variable into a categorical variable
data.ratio $ecoregion <- as.factor(data.ratio $ecoregion)

###MULTIPLE IMPUTATION-------------------#use mice to impute the SEs that we are missing
mice.dat <- data.ratio[, c('mort.ratio', 'se.combo', 'delta.cutin', 'Project.x', 'n.turbines', 'Nights', 'RD')]
predMatrix <- make.predictorMatrix(mice.dat)
predMatrix[c(1,3,4,5,6,7), ] <- 0
impMethod <- make.method(mice.dat)
imp <- mice(mice.dat, m=50, predictorMatrix = predMatrix, method = impMethod, seed = 3)
se.imp <- matrix(NA, nrow = nrow(data.ratio), ncol = 50)

#run the newly imputed data through the model fit process
####fm0 no mods####
mod.sum <- aicc.sum <- list()

for(n in 1:ncol(se.imp)){
 imp.tmp <- complete(imp, n)
 data.ratio$se.combo <- imp.tmp$se.combo

#build basic structure of variance-covariance matrix for the study
 ratio.vcov <- matrix(NA, nrow = nrow(data.ratio), ncol = nrow(data.ratio))
 diag(ratio.vcov) <- data.ratio$se.combo

#estimate uncertainty for sites that share controls
#identify controls for each site (here I pulled this information from a previous database)

data.ratio$ctrl.id <- c(1,2,3,4,5,6,7,8,9,10,11,12,13,13,14,10,15,15,16,16,17,18,19,20,20,21,21,22,22,23,23,24,24,25,25,25)
 dup.ctrl <- c(10, 13, 15, 16, 20, 21, 22, 23, 24, 25)

#calculate the component of RR sampling variance from the control for each ctrl-trt pairing
ctrl.effsize <- (data.ratio$cont.se)^2/(data.ratio$cont.bats.th)^2

#calculate the pairwise correlation between all ctrl-trt pairings
#pairs that share a control gets the calculated uncertainty between both RRs (SE1*SE2) while all other
#pairings get a 0 then the control variance component is divided by the combined uncertainty for the RR
 RR.secombos <- matrix(NA, nrow = nrow(ratio.vcov), ncol = ncol(ratio.vcov))
 cor.mat <- matrix(NA, nrow = nrow(ratio.vcov), ncol = ncol(ratio.vcov))
 for(i in 1:nrow(RR.secombos)){
 for(j in 1:ncol(RR.secombos)){
 if(data.ratio$ctrl.id[i] == data.ratio$ctrl.id[j] & data.ratio$ctrl.id[i] %in% dup.ctrl & data.ratio$ctrl.id[j] %in% dup.ctrl){
 RR.secombos[i, j] <- data.ratio$se.combo[i]*data.ratio$se.combo[j]
 cor.mat[i, j] <- ctrl.effsize[i]/RR.secombos[i, j]
 } else{
 RR.secombos[i, j] <- 0
 cor.mat[i, j] <- 0
 }
 }
 }

#main diagonal of the correlation matrix to 1
 diag(cor.mat) <- 1
#add study var to the RRsecombos
 diag(RR.secombos) <- data.ratio$se.combo
 V <- RR.secombos

#no moderators (for overall effect size)
 nomod <- rma.mv(yi=log(mort.ratio), V=V, random=~1|Project.x, data=data.ratio, method="ML")
 mod.sum[[n]] <- nomod
 aicc.sum[[n]] <- AICc(nomod)
}

fm0.imp <- pool(mod.sum)
fm0.mira <- as.mira(mod.sum)
fm0.aicc <- unlist(aicc.sum)

###CATEGORICAL MODELS-------------
####fm1 1|Site + ctrl.cutin####
mod.sum <- list()
for(n in 1:ncol(se.imp)){
 imp.tmp <- complete(imp, n)
 data.ratio$se.combo <- imp.tmp$se.combo
#build basic structure of variance-covariance matrix for the study
 ratio.vcov <- matrix(NA, nrow = nrow(data.ratio), ncol = nrow(data.ratio))
 diag(ratio.vcov) <- data.ratio$se.combo
 #estimate uncertainty for sites that share controls
 #identify controls for each site (here I pulled this information from a previous database)
 data.ratio$ctrl.id <- c(1,2,3,4,5,6,7,8,9,10,11,12,13,13,14,10,15,15,16,16,17,18,19,20,20,21,21,22,22,23,23,24,24,25,25,25)
 dup.ctrl <- c(10, 13, 15, 16, 20, 21, 22, 23, 24, 25)
 #calculate the component of RR sampling variance from the control for each ctrl-trt pairing
 ctrl.effsize <- (data.ratio$cont.se)^2/(data.ratio$cont.bats.th)^2
 #calculate the pairwise correlation between all ctrl-trt pairings
 #pairs that share a control gets the calculated uncertainty between both RRs (SE1*SE2) while all other
 #pairings get a 0
 #then the control variance component is divided by the combined uncertainty for the RRs
 RR.secombos <- matrix(NA, nrow = nrow(ratio.vcov), ncol = ncol(ratio.vcov)
 cor.mat <- matrix(NA, nrow = nrow(ratio.vcov), ncol = ncol(ratio.vcov)
 for(i in 1:nrow(RR.secombos)){
 for(j in 1:ncol(RR.secombos)){
 if(data.ratio$ctrl.id[i] == data.ratio$ctrl.id[j] & data.ratio$ctrl.id[i] %in% dup.ctrl & data.ratio$ctrl.id[j] %in% dup.ctrl){
 RR.secombos[i, j] <- data.ratio$se.combo[i]*data.ratio$se.combo[j]
 cor.mat[i, j] <- ctrl.effsize[i]/RR.secombos[i, j]
 } else{
 RR.secombos[i, j] <- 0
 cor.mat[i, j] <- 0
 }
 }
 }

#main diagonal of the correlation matrix to 1
 diag(cor.mat) <- 1
 #add study var to the RRsecombos
 diag(RR.secombos) <- data.ratio$se.combo
 V <- RR.secombos
 fm1.fixed <- rma.mv(yi=log(mort.ratio), V=V, mods=~scale.control.cutin, random=~1|Project.x, data=data.ratio, method="ML")
 mod.sum[[n]] <- fm1.fixed
 aicc.sum[[n]] <- AICc(fm1.fixed)
}
fm1.imp <- pool(mod.sum)
fm1.mira <- as.mira(mod.sum)
fm1.aicc <- unlist(aicc.sum)

####fm2 | #delta cut in + control + 1|site####
mod.sum <- list()
for(n in 1:ncol(se.imp)){
 imp.tmp <- complete(imp, n)
 data.ratio$se.combo <- imp.tmp$se.combo
 #build basic structure of variance-covariance matrix for the study
 ratio.vcov <- matrix(NA, nrow = nrow(data.ratio), ncol = nrow(data.ratio))
 diag(ratio.vcov) <- data.ratio$se.combo
 #estimate uncertainty for sites that share controls
 #identify controls for each site (here I pulled this information from a previous database)
 data.ratio$ctrl.id <- c(1,2,3,4,5,6,7,8,9,10,11,12,13,13,14,10,15,15,16,16,17,18,19,20,20,21,21,22,22,23,23,24,24,25,25,25)
 dup.ctrl <- c(10, 13, 15, 16, 20, 21, 22, 23, 24, 25)
 #calculate the component of RR sampling variance from the control for each ctrl-trt pairing
 ctrl.effsize <- (data.ratio$cont.se)^2/(data.ratio$cont.bats.th)^2
 #calculate the pairwise correlation between all ctrl-trt pairings
 #pairs that share a control gets the calculated uncertainty between both RRs (SE1*SE2) while all other pairings get a 0
 #then the control variance component is divided by the combined uncertainty for the RRs
 RR.secombos <- matrix(NA, nrow = nrow(ratio.vcov), ncol = ncol(ratio.vcov))
 cor.mat <- matrix(NA, nrow = nrow(ratio.vcov), ncol = ncol(ratio.vcov))
 for(i in 1:nrow(RR.secombos)){
 for(j in 1:ncol(RR.secombos)){
 if(data.ratio$ctrl.id[i] == data.ratio$ctrl.id[j] & data.ratio$ctrl.id[i] %in% dup.ctrl & data.ratio$ctrl.id[j] %in% dup.ctrl){
 RR.secombos[i, j] <- data.ratio$se.combo[i]*data.ratio$se.combo[j]
 cor.mat[i, j] <- ctrl.effsize[i]/RR.secombos[i, j]
 } else{
 RR.secombos[i, j] <- 0
 cor.mat[i, j] <- 0
 }
 }
 }
 #main diagonal of the correlation matrix to 1
 diag(cor.mat) <- 1
 #add study var to the RRsecombos
 diag(RR.secombos) <- data.ratio$se.combo
 V <- RR.secombos
 #model with site and control cut-in and delta.cut.in
 fm2.fixed <- rma.mv(yi=log(mort.ratio), V=V, mods=~scale.control.cutin + delta.cutin.cat, random=~1|Project.x, data=data.ratio, method="ML")
 mod.sum[[n]] <- fm2.fixed
 aicc.sum[[n]] <- AICc(fm2.fixed)
}
fm2.imp <- pool(mod.sum)
fm2.mira <- as.mira(mod.sum)
fm2.aicc <- unlist(aicc.sum)

####fm3 delta cutin cat + control cutin + ecoregion + 1|project####
mod.sum <- list()
for(n in 1:ncol(se.imp)){
 imp.tmp <- complete(imp, n)
 data.ratio$se.combo <- imp.tmp$se.combo
 #build basic structure of variance-covariance matrix for the study
 ratio.vcov <- matrix(NA, nrow = nrow(data.ratio), ncol = nrow(data.ratio))
 diag(ratio.vcov) <- data.ratio$se.combo
 #estimate uncertainty for sites that share controls
 #identify controls for each site (here I pulled this information from a previous database) data.ratio$ctrl.id <- c(1,2,3,4,5,6,7,8,9,10,11,12,13,13,14,10,15,15,16,16,17,18,19,20,20,21,21,22,22,23,23,24,24,25,25,25)
 dup.ctrl <- c(10, 13, 15, 16, 20, 21, 22, 23, 24, 25)
 #calculate the component of RR sampling variance from the control for each ctrl-trt pairing
 ctrl.effsize <- (data.ratio$cont.se)^2/(data.ratio$cont.bats.th)^2
 #calculate the pairwise correlation between all ctrl-trt pairing
 #pairs that share a control gets the calculated uncertainty between both RRs (SE1*SE2) while all other pairings get a 0
 #then the control variance component is divided by the combined uncertainty for the RRs
 RR.secombos <- matrix(NA, nrow = nrow(ratio.vcov), ncol = ncol(ratio.vcov))
 cor.mat <- matrix(NA, nrow = nrow(ratio.vcov), ncol = ncol(ratio.vcov))
 for(i in 1:nrow(RR.secombos)){
 for(j in 1:ncol(RR.secombos)){
 if(data.ratio$ctrl.id[i] == data.ratio$ctrl.id[j] & data.ratio$ctrl.id[i] %in% dup.ctrl & data.ratio$ctrl.id[j] %in% dup.ctrl){
 RR.secombos[i, j] <- data.ratio$se.combo[i]*data.ratio$se.combo[j]
 cor.mat[i, j] <- ctrl.effsize[i]/RR.secombos[i, j]
 } else{
 RR.secombos[i, j] <- 0
 cor.mat[i, j] <- 0
 }
 }
 }
 #main diagonal of the correlation matrix to 1
 diag(cor.mat) <- 1
 #add study var to the RRsecombos
 diag(RR.secombos) <- data.ratio$se.combo
 V <- RR.secombos
 #model with site and control cut-in and delta.cut.in
 fm3.fixed <- rma.mv(yi=log(mort.ratio), V=V, mods=~delta.cutin.cat + ecoregion+scale.control.cutin, random=~1|Project.x, data=data.ratio, method="ML")
 mod.sum[[n]] <- fm3.fixed
 aicc.sum[[n]] <- AICc(fm3.fixed)
}
fm3.imp <- pool(mod.sum)
fm3.mira <- as.mira(mod.sum)
fm3.aicc <- unlist(aicc.sum)

####fm4 delta cutin cat + control cutin + rd + 1|project####
mod.sum <- list()
for(n in 1:ncol(se.imp)){
 imp.tmp <- complete(imp, n)
 data.ratio$se.combo <- imp.tmp$se.combo
 #build basic structure of variance-covariance matrix for the study
 ratio.vcov <- matrix(NA, nrow = nrow(data.ratio), ncol = nrow(data.ratio))
 diag(ratio.vcov) <- data.ratio$se.combo
 #estimate uncertainty for sites that share controls
 #identify controls for each site (here I pulled this information from a previous database)
 data.ratio$ctrl.id <- c(1,2,3,4,5,6,7,8,9,10,11,12,13,13,14,10,15,15,16,16,17,18,19,20,20,21,21,22,22,23,23,24,24,25,25,25)
 dup.ctrl <- c(10, 13, 15, 16, 20, 21, 22, 23, 24, 25)
 #calculate the component of RR sampling variance from the control for each ctrl-trt pairing
 ctrl.effsize <- (data.ratio$cont.se)^2/(data.ratio$cont.bats.th)^2
 #calculate the pairwise correlation between all ctrl-trt pairings
 #pairs that share a control gets the calculated uncertainty between both RRs (SE1*SE2) while all other pairings get a 0
 #then the control variance component is divided by the combined uncertainty for the RRs
 RR.secombos <- matrix(NA, nrow = nrow(ratio.vcov), ncol = ncol(ratio.vcov))
 cor.mat <- matrix(NA, nrow = nrow(ratio.vcov), ncol = ncol(ratio.vcov))
 for(i in 1:nrow(RR.secombos)){
 for(j in 1:ncol(RR.secombos)){
 if(data.ratio$ctrl.id[i] == data.ratio$ctrl.id[j] & data.ratio$ctrl.id[i] %in% dup.ctrl & data.ratio$ctrl.id[j] %in% dup.ctrl){
 RR.secombos[i, j] <- data.ratio$se.combo[i]*data.ratio$se.combo[j]
 cor.mat[i, j] <- ctrl.effsize[i]/RR.secombos[i, j]
 } else{
 RR.secombos[i, j] <- 0
 cor.mat[i, j] <- 0
 }
 }
 }
 #main diagonal of the correlation matrix to 1
 diag(cor.mat) <- 1
 #add study var to the RRsecombos
 diag(RR.secombos) <- data.ratio$se.combo
 V <- RR.secombos
 #model with site and control cut-in and delta.cut.in
 fm4.fixed <- rma.mv(yi=log(mort.ratio), V=V, mods=~delta.cutin.cat + scale.rd+scale.control.cutin, random=~1|Project.x, data=data.ratio, method="ML")
 mod.sum[[n]] <- fm4.fixed
 aicc.sum[[n]] <- AICc(fm4.fixed)
}
fm4.imp <- pool(mod.sum)
fm4.mira <- as.mira(mod.sum)
fm4.aicc <- unlist(aicc.sum)

####fm5 delta cutin cat + control cutin + rd + ecoregion+ 1|project####
mod.sum <- list()
for(n in 1:ncol(se.imp)){
 imp.tmp <- complete(imp, n)
 data.ratio$se.combo <- imp.tmp$se.combo
 #build basic structure of variance-covariance matrix for the study
 ratio.vcov <- matrix(NA, nrow = nrow(data.ratio), ncol = nrow(data.ratio))
 diag(ratio.vcov) <- data.ratio$se.combo
 #estimate uncertainty for sites that share controls
 #identify controls for each site (here I pulled this information from a previous database
 data.ratio$ctrl.id <- c(1,2,3,4,5,6,7,8,9,10,11,12,13,13,14,10,15,15,16,16,17,18,19,20,20,21,21,22,22,23,23,24,24,25,25,25)
 dup.ctrl <- c(10, 13, 15, 16, 20, 21, 22, 23, 24, 25)
 #calculate the component of RR sampling variance from the control for each ctrl-trt pairing
 ctrl.effsize <- (data.ratio$cont.se)^2/(data.ratio$cont.bats.th)^2
 #calculate the pairwise correlation between all ctrl-trt pairings
 #pairs that share a control gets the calculated uncertainty between both RRs (SE1*SE2) while all other pairings get a 0
 #then the control variance component is divided by the combined uncertainty for the RRs
 RR.secombos <- matrix(NA, nrow = nrow(ratio.vcov), ncol = ncol(ratio.vcov))
 cor.mat <- matrix(NA, nrow = nrow(ratio.vcov), ncol = ncol(ratio.vcov))
 for(i in 1:nrow(RR.secombos)){
 for(j in 1:ncol(RR.secombos)){
 if(data.ratio$ctrl.id[i] == data.ratio$ctrl.id[j] & data.ratio$ctrl.id[i] %in% dup.ctrl & data.ratio$ctrl.id[j] %in% dup.ctrl){
 RR.secombos[i, j] <- data.ratio$se.combo[i]*data.ratio$se.combo[j]
 cor.mat[i, j] <- ctrl.effsize[i]/RR.secombos[i, j]
 } else{
 RR.secombos[i, j] <- 0
 cor.mat[i, j] <- 0
 }
 }
 }
 #main diagonal of the correlation matrix to 1
 diag(cor.mat) <- 1
 #add study var to the RRsecombos
 diag(RR.secombos) <- data.ratio$se.combo
 V <- RR.secombos
 fm5.fixed <- rma.mv(yi=log(mort.ratio), V=V, mods=~delta.cutin.cat + scale.rd+scale.control.cutin+ecoregion, random=~1|Project.x, data=data.ratio, method="ML")
 mod.sum[[n]] <- fm5.fixed
 aicc.sum[[n]] <- AICc(fm5.fixed)
}
fm5.imp <- pool(mod.sum)
fm5.mira <- as.mira(mod.sum)
fm5.aicc <- unlist(aicc.sum)

###CONTINUOUS MODELS----
####fm6 delta cut in + control + project as random####
mod.sum <- list()
for(n in 1:ncol(se.imp)){
 imp.tmp <- complete(imp, n)
 data.ratio$se.combo <- imp.tmp$se.combo
 #build basic structure of variance-covariance matrix for the study
 ratio.vcov <- matrix(NA, nrow = nrow(data.ratio), ncol = nrow(data.ratio))
 diag(ratio.vcov) <- data.ratio$se.combo
 #estimate uncertainty for sites that share controls
 #identify controls for each site (here I pulled this information from a previous database)
 data.ratio$ctrl.id <- c(1,2,3,4,5,6,7,8,9,10,11,12,13,13,14,10,15,15,16,16,17,18,19,20,20,21,21,22,22,23,23,24,24,25,25,25)
 dup.ctrl <- c(10, 13, 15, 16, 20, 21, 22, 23, 24, 25)
 #calculate the component of RR sampling variance from the control for each ctrl-trt pairing
 ctrl.effsize <- (data.ratio$cont.se)^2/(data.ratio$cont.bats.th)^2
 #calculate the pairwise correlation between all ctrl-trt pairings
 #pairs that share a control gets the calculated uncertainty between both RRs (SE1*SE2) while all other pairings get a 0
 #then the control variance component is divided by the combined uncertainty for the RR
 RR.secombos <- matrix(NA, nrow = nrow(ratio.vcov), ncol = ncol(ratio.vcov))
 cor.mat <- matrix(NA, nrow = nrow(ratio.vcov), ncol = ncol(ratio.vcov))
 for(i in 1:nrow(RR.secombos)){
 for(j in 1:ncol(RR.secombos)){
 if(data.ratio$ctrl.id[i] == data.ratio$ctrl.id[j] & data.ratio$ctrl.id[i] %in% dup.ctrl & data.ratio$ctrl.id[j] %in% dup.ctrl){
 RR.secombos[i, j] <- data.ratio$se.combo[i]*data.ratio$se.combo[j]
 cor.mat[i, j] <- ctrl.effsize[i]/RR.secombos[i, j]
 } else{
 RR.secombos[i, j] <- 0
 cor.mat[i, j] <- 0
 }
 }
 }
 #main diagonal of the correlation matrix to 1
 diag(cor.mat) <- 1
 #add study var to the RRsecombos
 diag(RR.secombos) <- data.ratio$se.combo
 V <- RR.secombos
 fm6.fixed <- rma.mv(yi=log(mort.ratio), V=V, mods=~scale.delta.cutin + scale.control.cutin, random=~1|Project.x, data=data.ratio, method="ML")
 mod.sum[[n]] <- fm6.fixed
 aicc.sum[[n]] <- AICc(fm6.fixed)
}
fm6.imp <- pool(mod.sum)
fm6.mira <- as.mira(mod.sum)
fm6.aicc <- unlist(aicc.sum)

####fm7 delta cutin cat + control cutin + ecoregion + 1|project####
mod.sum <- list()
for(n in 1:ncol(se.imp)){
 imp.tmp <- complete(imp, n)
 data.ratio$se.combo <- imp.tmp$se.combo
 #build basic structure of variance-covariance matrix for the study
 ratio.vcov <- matrix(NA, nrow = nrow(data.ratio), ncol = nrow(data.ratio))
 diag(ratio.vcov) <- data.ratio$se.combo
 #estimate uncertainty for sites that share controls
 #identify controls for each site (here I pulled this information from a previous database)
 data.ratio$ctrl.id <- c(1,2,3,4,5,6,7,8,9,10,11,12,13,13,14,10,15,15,16,16,17,18,19,20,20,21,21,22,22,23,23,24,24,25,25,25)
 dup.ctrl <- c(10, 13, 15, 16, 20, 21, 22, 23, 24, 25)
 #calculate the component of RR sampling variance from the control for each ctrl-trt pairing
 ctrl.effsize <- (data.ratio$cont.se)^2/(data.ratio$cont.bats.th)^2
 #calculate the pairwise correlation between all ctrl-trt pairings

#pairs that share a control gets the calculated uncertainty between both RRs (SE1*SE2) while all other pairings get a 0
 #then the control variance component is divided by the combined uncertainty for the RRs
 RR.secombos <- matrix(NA, nrow = nrow(ratio.vcov), ncol = ncol(ratio.vcov))
 cor.mat <- matrix(NA, nrow = nrow(ratio.vcov), ncol = ncol(ratio.vcov))
 for(i in 1:nrow(RR.secombos)){
 for(j in 1:ncol(RR.secombos)){
 if(data.ratio$ctrl.id[i] == data.ratio$ctrl.id[j] & data.ratio$ctrl.id[i] %in% dup.ctrl & data.ratio$ctrl.id[j] %in% dup.ctrl){
 RR.secombos[i, j] <- data.ratio$se.combo[i]*data.ratio$se.combo[j]
 cor.mat[i, j] <- ctrl.effsize[i]/RR.secombos[i, j]
 } else{
 RR.secombos[i, j] <- 0
 cor.mat[i, j] <- 0
 }
 }
 }
 #main diagonal of the correlation matrix to 1
 diag(cor.mat) <- 1
 #add study var to the RRsecombos
 diag(RR.secombos) <- data.ratio$se.combo
 V <- RR.secombos
 fm7.fixed <- rma.mv(yi=log(mort.ratio), V=V, mods=~scale.delta.cutin + ecoregion+scale.control.cutin, random=~1|Project.x, data=data.ratio, method="ML")
 mod.sum[[n]] <- fm7.fixed
 aicc.sum[[n]] <- AICc(fm7.fixed)
}
fm7.imp <- pool(mod.sum)
fm7.mira <- as.mira(mod.sum)
fm7.aicc <- unlist(aicc.sum)

####fm8 delta cutin cat + control cutin + rd + 1|project####
mod.sum <- list()
for(n in 1:ncol(se.imp)){
 imp.tmp <- complete(imp, n)
 data.ratio$se.combo <- imp.tmp$se.combo
 #build basic structure of variance-covariance matrix for the study
 ratio.vcov <- matrix(NA, nrow = nrow(data.ratio), ncol = nrow(data.ratio))
 diag(ratio.vcov) <- data.ratio$se.combo
 #estimate uncertainty for sites that share controls
 #identify controls for each site (here I pulled this information from a previous database)
 data.ratio$ctrl.id <- c(1,2,3,4,5,6,7,8,9,10,11,12,13,13,14,10,15,15,16,16,17,18,19,20,20,21,21,22,22,23,23,24,24,25,25,25)
 dup.ctrl <- c(10, 13, 15, 16, 20, 21, 22, 23, 24, 25)
 #calculate the component of RR sampling variance from the control for each ctrl-trt pairing
 ctrl.effsize <- (data.ratio$cont.se)^2/(data.ratio$cont.bats.th)^2
 #calculate the pairwise correlation between all ctrl-trt pairings
 #pairs that share a control gets the calculated uncertainty between both RRs (SE1*SE2) while all other pairings get a 0
 #then the control variance component is divided by the combined uncertainty for the RRs
 RR.secombos <- matrix(NA, nrow = nrow(ratio.vcov), ncol = ncol(ratio.vcov))
 cor.mat <- matrix(NA, nrow = nrow(ratio.vcov), ncol = ncol(ratio.vcov))
 for(i in 1:nrow(RR.secombos)){
 for(j in 1:ncol(RR.secombos)){
 if(data.ratio$ctrl.id[i] == data.ratio$ctrl.id[j] & data.ratio$ctrl.id[i] %in% dup.ctrl & data.ratio$ctrl.id[j] %in% dup.ctrl){
 RR.secombos[i, j] <- data.ratio$se.combo[i]*data.ratio$se.combo[j]
 cor.mat[i, j] <- ctrl.effsize[i]/RR.secombos[i, j]
 } else{
 RR.secombos[i, j] <- 0
 cor.mat[i, j] <- 0
 }
 }
 }
 #main diagonal of the correlation matrix to 1
 diag(cor.mat) <- 1
 #add study var to the RRsecombos
 diag(RR.secombos) <- data.ratio$se.combo
 V <- RR.secombos
 fm8.fixed <- rma.mv(yi=log(mort.ratio), V=V, mods=~scale.delta.cutin + scale.rd+scale.control.cutin, random=~1|Project.x, data=data.ratio, method="ML")
 mod.sum[[n]] <- fm8.fixed
 aicc.sum[[n]] <- AICc(fm8.fixed)
}
fm8.imp <- pool(mod.sum)
fm8.mira <- as.mira(mod.sum)
fm8.aicc <- unlist(aicc.sum)

####fm9 delta cutin cat + control cutin + rd +ecoregion + 1|project####
mod.sum <- list()
for(n in 1:ncol(se.imp)){
 imp.tmp <- complete(imp, n)
 data.ratio$se.combo <- imp.tmp$se.combo
 #build basic structure of variance-covariance matrix for the study
 ratio.vcov <- matrix(NA, nrow = nrow(data.ratio), ncol = nrow(data.ratio))
 diag(ratio.vcov) <- data.ratio$se.combo
 #estimate uncertainty for sites that share controls
 #identify controls for each site (here I pulled this information from a previous database)
 data.ratio$ctrl.id <- c(1,2,3,4,5,6,7,8,9,10,11,12,13,13,14,10,15,15,16,16,17,18,19,20,20,21,21,22,22,23,23,24,24,25,25,25)
 dup.ctrl <- c(10, 13, 15, 16, 20, 21, 22, 23, 24, 25)
 #calculate the component of RR sampling variance from the control for each ctrl-trt pairing
 ctrl.effsize <- (data.ratio$cont.se)^2/(data.ratio$cont.bats.th)^2
 #calculate the pairwise correlation between all ctrl-trt pairings
 #pairs that share a control gets the calculated uncertainty between both RRs (SE1*SE2) while all other pairings get a 0
 #then the control variance component is divided by the combined uncertainty for the RRs
 RR.secombos <- matrix(NA, nrow = nrow(ratio.vcov), ncol = ncol(ratio.vcov))
 cor.mat <- matrix(NA, nrow = nrow(ratio.vcov), ncol = ncol(ratio.vcov))
 for(i in 1:nrow(RR.secombos)){
 for(j in 1:ncol(RR.secombos)){
 if(data.ratio$ctrl.id[i] == data.ratio$ctrl.id[j] & data.ratio$ctrl.id[i] %in% dup.ctrl & data.ratio$ctrl.id[j] %in% dup.ctrl){
 RR.secombos[i, j] <- data.ratio$se.combo[i]*data.ratio$se.combo[j]
 cor.mat[i, j] <- ctrl.effsize[i]/RR.secombos[i, j]
 } else{
 RR.secombos[i, j] <- 0
 cor.mat[i, j] <- 0
 }
 }
 }
 #main diagonal of the correlation matrix to 1
 diag(cor.mat) <- 1
 #add study var to the RRsecombos
 diag(RR.secombos) <- data.ratio$se.combo
 V <- RR.secombos
 fm9.fixed <- rma.mv(yi=log(mort.ratio), V=V, mods=~scale.delta.cutin + scale.rd+scale.control.cutin+ecoregion, random=~1|Project.x, data=data.ratio, method="ML")
 mod.sum[[n]] <- fm9.fixed
 aicc.sum[[n]] <- AICc(fm9.fixed)
}
fm9.imp <- pool(mod.sum)
fm9.mira <- as.mira(mod.sum)
fm9.aicc <- unlist(aicc.sum)

###MODEL SELECTION--------------
####Pool AICc across imputations####
aicc.comb <- list(fm1.aicc, fm2.aicc, fm3.aicc, fm4.aicc, fm5.aicc, fm6.aicc, fm7.aicc, fm8.aicc, fm9.aicc)
aicc.table <- unlist(lapply(aicc.comb, mean))

#mean for each model
aic.all <- c(mean(fm1.aicc), mean(fm2.aicc), mean(fm3.aicc), mean(fm4.aicc), mean(fm5.aicc), mean(fm6.aicc), mean(fm7.aicc), mean(fm8.aicc), mean(fm9.aicc))
aic.all <- as.data.frame(aic.all)
aic.all$model <- c("fm1", "fm2", "fm3", "fm4", "fm5", "fm6", "fm7", "fm8", "fm9")
weights <- akaike.weights(aic.all$aic.all)
aic.all$deltaaic <- weights$deltaAIC
aic.all$relLL <- weights$rel.LL
aic.all$weights <- weights$weights

####pooled -loglik values####
library(foreach)
fm1.loglik <- list()
fm1.loglik <- foreach(i = 1:50) %do% {
 loglik <- as.data.frame(logLik(fm1.mira$analyses[[i]]))}
fm1.loglik <- as.data.frame(dplyr::bind_rows(fm1.loglik))
mean(fm1.loglik)

fm2.loglik <- list()
fm2.loglik <- foreach(i = 1:50) %do% {
 loglik <- as.data.frame(logLik(fm2.mira$analyses[[i]]))}
fm2.loglik <- dplyr::bind_rows(fm2.loglik)

fm3.loglik <- list()
fm3.loglik <- foreach(i = 1:50) %do% {
 loglik <- as.data.frame(logLik(fm3.mira$analyses[[i]]))}
fm3.loglik <- dplyr::bind_rows(fm3.loglik)

fm4.loglik <- list()
fm4.loglik <- foreach(i = 1:50) %do% {
 loglik <- as.data.frame(logLik(fm4.mira$analyses[[i]]))}
fm4.loglik <- dplyr::bind_rows(fm4.loglik)

fm5.loglik <- list()
fm5.loglik <- foreach(i = 1:50) %do% {
 loglik <- as.data.frame(logLik(fm5.mira$analyses[[i]]))}
fm5.loglik <- dplyr::bind_rows(fm5.loglik)

fm6.loglik <- list()
fm6.loglik <- foreach(i = 1:50) %do% {
 loglik <- as.data.frame(logLik(fm6.mira$analyses[[i]]))}
fm6.loglik <- dplyr::bind_rows(fm6.loglik)

fm7.loglik <- list()
fm7.loglik <- foreach(i = 1:50) %do% {
 loglik <- as.data.frame(logLik(fm7.mira$analyses[[i]]))}
fm7.loglik <- dplyr::bind_rows(fm7.loglik)

fm8.loglik <- list()
fm8.loglik <- foreach(i = 1:50) %do% {
 loglik <- as.data.frame(logLik(fm8.mira$analyses[[i]]))}
fm8.loglik <- dplyr::bind_rows(fm8.loglik)

fm9.loglik <- list()
fm9.loglik <- foreach(i = 1:50) %do% {
 loglik <- as.data.frame(logLik(fm9.mira$analyses[[i]]))}
fm9.loglik <- dplyr::bind_rows(fm9.loglik)

loglik.all <- c(mean(fm1.loglik$`c(x)`), mean(fm2.loglik$`c(x)`), mean(fm3.loglik$`c(x)`),
 mean(fm4.loglik$`c(x)`), mean(fm5.loglik$`c(x)`), mean(fm6.loglik$`c(x)`), mean(fm7.loglik$`c(x)`), mean(fm8.loglik$`c(x)`), mean(fm9.loglik$`c(x)`))
aic.all$loglik <- loglik.all

####Pooled Cook's Distance####
fm1.cooksd <- list()
fm1.cooksd <- foreach(i = 1:50) %do% {
 cooksd<- as.data.frame(cooks.distance.rma.mv(fm1.mira$analyses[[i]]))
}
fm1.cooksd <- dplyr::bind_rows(fm1.cooksd)
fm1.cooksd <- as.data.frame(fm1.cooksd)
fm1.cooksd$study <- rep(1:36, 50)
colnames(fm1.cooksd) <- c("cooksd", "study")

fm2.cooksd <- list()
fm2.cooksd <- foreach(i = 1:50) %do% {
 cooksd<- as.data.frame(cooks.distance.rma.mv(fm2.mira$analyses[[i]]))
}

fm2.cooksd <- dplyr::bind_rows(fm2.cooksd)
fm2.cooksd <- as.data.frame(fm2.cooksd)
fm2.cooksd$study <- rep(1:36, 50)
colnames(fm2.cooksd) <- c("cooksd", "study")

fm3.cooksd <- list()
fm3.cooksd <- foreach(i = 1:50) %do% {
 cooksd<- as.data.frame(cooks.distance.rma.mv(fm3.mira$analyses[[i]]))
}
fm3.cooksd <- dplyr::bind_rows(fm3.cooksd)
fm3.cooksd <- as.data.frame(fm3.cooksd)
fm3.cooksd$study <- rep(1:36, 50)
colnames(fm3.cooksd) <- c("cooksd", "study")

fm4.cooksd <- list()
fm4.cooksd <- foreach(i = 1:50) %do% {
 cooksd<- as.data.frame(cooks.distance.rma.mv(fm4.mira$analyses[[i]]))
}

fm4.cooksd <- dplyr::bind_rows(fm4.cooksd)
fm4.cooksd <- as.data.frame(fm4.cooksd)
fm4.cooksd$study <- rep(1:36, 50)
colnames(fm4.cooksd) <- c("cooksd", "study")

fm5.cooksd <- list()
fm5.cooksd <- foreach(i = 1:50) %do% {
 cooksd<- as.data.frame(cooks.distance.rma.mv(fm5.mira$analyses[[i]]))
}
fm5.cooksd <- dplyr::bind_rows(fm5.cooksd)
fm5.cooksd <- as.data.frame(fm5.cooksd)
fm5.cooksd$study <- rep(1:36, 50)
colnames(fm5.cooksd) <- c("cooksd", "study")

fm6.cooksd <- list()
fm6.cooksd <- foreach(i = 1:50) %do% {
 cooksd<- as.data.frame(cooks.distance.rma.mv(fm6.mira$analyses[[i]]))
}
fm6.cooksd <- dplyr::bind_rows(fm6.cooksd)
fm6.cooksd <- as.data.frame(fm6.cooksd)
fm6.cooksd$study <- rep(1:36, 50)
colnames(fm6.cooksd) <- c("cooksd", "study")

fm7.cooksd <- list()
fm7.cooksd <- foreach(i = 1:50) %do% {
 cooksd<- as.data.frame(cooks.distance.rma.mv(fm7.mira$analyses[[i]]))
}
fm7.cooksd <- dplyr::bind_rows(fm7.cooksd)
fm7.cooksd <- as.data.frame(fm7.cooksd)
fm7.cooksd$study <- rep(1:36, 50)
colnames(fm7.cooksd) <- c("cooksd", "study")

fm8.cooksd <- list()
fm8.cooksd <- foreach(i = 1:50) %do% {
 cooksd<- as.data.frame(cooks.distance.rma.mv(fm8.mira$analyses[[i]]))
}
fm8.cooksd <- dplyr::bind_rows(fm8.cooksd)
fm8.cooksd <- as.data.frame(fm8.cooksd)
fm8.cooksd$study <- rep(1:36, 50)
colnames(fm8.cooksd) <- c("cooksd", "study")

fm9.cooksd <- list()
fm9.cooksd <- foreach(i = 1:50) %do% {
 cooksd<- as.data.frame(cooks.distance.rma.mv(fm9.mira$analyses[[i]]))
}
fm9.cooksd <- dplyr::bind_rows(fm9.cooksd)
fm9.cooksd <- as.data.frame(fm9.cooksd)
fm9.cooksd$study <- rep(1:36, 50)
colnames(fm9.cooksd) <- c("cooksd", "study")

fm1.cooksd$study <- as.factor(fm1.cooksd$study)
fm1.cooksd.sum <- summary_by(cooksd~study, data=fm1.cooksd, FUN=mean)
fm2.cooksd$study <- as.factor(fm2.cooksd$study)
fm2.cooksd.sum <- summary_by(cooksd~study, data=fm2.cooksd, FUN=mean)
fm3.cooksd$study <- as.factor(fm3.cooksd$study)
fm3.cooksd.sum <- summary_by(cooksd~study, data=fm3.cooksd, FUN=mean)
fm4.cooksd$study <- as.factor(fm4.cooksd$study)
fm4.cooksd.sum <- summary_by(cooksd~study, data=fm4.cooksd, FUN=mean)
fm5.cooksd$study <- as.factor(fm5.cooksd$study)
fm5.cooksd.sum <- summary_by(cooksd~study, data=fm5.cooksd, FUN=mean)
fm6.cooksd$study <- as.factor(fm6.cooksd$study)
fm6.cooksd.sum <- summary_by(cooksd~study, data=fm6.cooksd, FUN=mean)
fm7.cooksd$study <- as.factor(fm7.cooksd$study)
fm7.cooksd.sum <- summary_by(cooksd~study, data=fm7.cooksd, FUN=mean)
fm8.cooksd$study <- as.factor(fm8.cooksd$study)
fm8.cooksd.sum <- summary_by(cooksd~study, data=fm8.cooksd, FUN=mean)
fm9.cooksd$study <- as.factor(fm9.cooksd$study)
fm9.cooksd.sum <- summary_by(cooksd~study, data=fm9.cooksd, FUN=mean)

####pooled heterogeneity####
fm2.results <- list()
fm2.results <- foreach(i = 1:50) %do% {
 QE<- fm2.mira$analyses[[i]]$QE
 QEp <- fm2.mira$analyses[[i]]$QEp
 sigma <- fm2.mira$analyses[[i]]$sigma2
 W <- solve(fm2.mira$analyses[[i]]$V)
 X <- model.matrix(fm2.mira$analyses[[i]])
 P <- W - W %*% X %*% solve(t(X) %*% W %*% X) %*% t(X) %*% W
 i2 <- 100 * fm2.mira$analyses[[i]]$sigma2 / (fm2.mira$analyses[[i]]$sigma2 + (fm2.mira$analyses[[i]]$k-fm2.mira$analyses[[i]]$p)/sum(diag(P)))
 return(as.data.frame(t(c(QE, QEp, sigma, i2))))
}
fm2.results <- dplyr::bind_rows(fm2.results)
fm2.results <- as.data.frame(fm2.results)

colnames(fm2.results) <- c("QE", "QEp", "sigma", "i2")mean(fm2.results$QE)
mean(fm2.results$QEp)
mean(fm2.results$sigma)
mean(fm2.results$i2)

fm6.results <- list()
fm6.results <- foreach(i = 1:50) %do% {
 QE<- fm6.mira$analyses[[i]]$QE
 QEp <- fm6.mira$analyses[[i]]$QEp
 sigma <- fm6.mira$analyses[[i]]$sigma2
 X <- model.matrix(fm6.mira$analyses[[i]])
 P <- W - W %*% X %*% solve(t(X) %*% W %*% X) %*% t(X) %*% W
 i2 <- 100 * fm6.mira$analyses[[i]]$sigma2 / (fm6.mira$analyses[[i]]$sigma2 + (fm6.mira$analyses[[i]]$k-fm6.mira$analyses[[i]]$p)/sum(diag(P)))
 return(as.data.frame(t(c(QE, QEp, sigma, i2))))
}
fm6.results <- dplyr::bind_rows(fm6.results)
fm6.results <- as.data.frame(fm6.results)
colnames(fm6.results) <- c("QE", "QEp", "sigma", "i2")
mean(fm6.results$QE)
mean(fm6.results$QEp)
mean(fm6.results$sigma)
mean(fm6.results$i2)

#Meta-analysis Power Analysis R Code

#Libraries used for analysis

library(broom)

library(ggplot2)

library(dplyr)

library(purrr)

library(doBy)

library(meta)

library(metafor)

library(fitdistrplus)

#This analysis uses the “data.ratio” dataset created in the above example (also Table 1).

data.ratio <- read.csv("Data/data.ratio_09302021.csv")

#fit a gamma distribution to the SE data so we can pull the shape and rate parameters from to conduct the simulation

sd.fit <- fitdist(data.ratio$se.combo[which(is.na(data.ratio$se.combo) == FALSE)], distr = 'gamma', method = 'mle')

#Simulation Function for the categorical model

#create a function that can simulate the data

metasim = function(

nrep = 5,

b0 = -0.75,

b1 = -0.43,

b2= -0.56,

sigma = 0.628,

shape = sd.fit$estimate[1],

rate = sd.fit$estimate[2]){

require(meta)

ngroup = 3

group = data.frame(delta.cutin = rep( c("A", "B", "C"), each = nrep))

eps = rnorm(ngroup*nrep, 0, sigma)

#ivw <- runif(ngroup*nrep, 0.21, 1.07) #values defined using the minimum and maxiumu combined SE values for all the data

ivw <- rgamma(ngroup*nrep, shape = shape, rate = rate) # new more scalable method for revision

mort = b0 + b1*(group == "B") + b2*(group=="C") + eps

fm.meta <- metagen(mort, ivw, data = group, comb.random = TRUE, comb.fixed = FALSE)

mortfit <- metareg(fm.meta, ~delta.cutin)

return(mortfit)

}

#b0, b1, and b2 are based on our current knowledge of the effect and can be altered to test other options

#Note that in order to test whether different from 1 (as 1 is effectively zero for the purposes of inference with a ratio), we have done 1-b0 for the b0 parameter #in the simulation

#Simulation Function for the continuous model

metasim = function(

nrep = 5,

b0 = -1.06,

b1 = -0.14,

sigma = 0.628,

shape = sd.fit$estimate[1],

rate = sd.fit$estimate[2]){

require(meta)

ngroup = 3

group = data.frame(group = rep( c("A", "B", "C"), each = nrep), dci = NA)

group$dci <- ifelse(group$group == 'A', sample(c(.5, 1, 1.3), nrep, replace = T), group$dci)

group$dci <- ifelse(group$group == 'B', sample(c(1.5, 1.9, 2.0, 2.3), nrep, replace = T), group$dci)

group$dci <- ifelse(group$group == 'C', sample(c(3, 3.5), nrep, replace = T), group$dci)

eps = rnorm(ngroup*nrep, 0, sigma)

#ivw <- runif(ngroup*nrep, 0.21, 1.07) #values defined using the minimum and maxiumu combined SE values for all the data

ivw <- rgamma(ngroup*nrep, shape = shape, rate = rate) # new more scalable method for revision mort = b0 + b1*group$dci + eps

mort = b0 + b1*group$dci + eps

fm.meta <- metagen(mort, ivw, data = group, comb.random = TRUE, comb.fixed = FALSE)

mortfit <- metareg(fm.meta, ~dci)

return(mortfit)

}

#Choosing either model type, you can run the simulation and extract the needed information for each model run

#Define number of simulations for all scenarios

nsims = 10000

#Scenario 1: The base scenario with 5 studies per category

sims.meta = rerun(nsims, metasim())

#extract the values that we want

sims.coeff1 <- matrix(unlist(lapply(sims.meta, function(x){x$b})), nrow = nsim, ncol = 3, byrow = TRUE)

sims.pval1 <- matrix(unlist(lapply(sims.meta, function(x){ifelse(x$pval > 0.05, 0, 1)})), nrow = nsim, ncol =

3, byrow = TRUE)

sims.i2.1 <- matrix(unlist(lapply(sims.meta, function (x) {x$I2})), nrow=nsim, ncol=1, byrow=TRUE)

sims.h2.1 <- matrix(unlist(lapply(sims.meta, function (x) {x$H2})), nrow=nsim, ncol=1, byrow=TRUE)

#This same code was run for all simulations, changing the number of studies (nrep=5,10,20,30) and beta values (see Table 2).

#Once all simulations are run, combine the data, and plot the results as needed

#end

Curtailment Study Power Analysis

#creating multiple functions for simulating data, conducting analysis, and summarizing the results

#background data simulation

curt.dat <- function(nsim, nturbines1, nturbines2, ndays, nhours, mort.i, diff, mean.cp, mean.se, search.i){

require(GenEst)

require(simsurv)

scenario <- data.frame(nsim = nsim, nturbines1 = nturbines1, nurtbines2 = nturbines2, ndays = ndays, nhours = nhours,

mort.i = mort.i, diff = diff, mean.cp = mean.cp, mean.se = mean.se, search.i = search.i)

#prepare arrays to store data from the simulations

trt_diff_sum <- list()

N1 <- array(NA, dim = c(nsim, nturbines1, ndays))

N2 <- array(NA, dim = c(nsim, nturbines2, ndays))

y1 <- array(NA, dim = c(nsim, nturbines1, ndays))

y2 <- array(NA, dim = c(nsim, nturbines2, ndays))

dat.list <- list()

#start the simulations

for(i in 1:nsim){

lambda1 <- mort.i #per turbine daily fatality rate

lambda2 <- lambda1 * diff #add in the treatment effect to adjust fatality for the curtailed turbines

#define the search.days

search.days <- seq(1, ndays, by = search.i)

#list turbines

turbs <- c(paste(1:nturbines1, 0, sep = "_"), paste(1:nturbines2, 1, sep = "_"))

#create the known carcass persistence probabilities

daily.cpp <- pexp(1:ndays + 1, 1/mean.cp, lower = FALSE, log = FALSE)

#simulate carcass persistence data for 50 carcasses

surv.exp <- simsurv('exponential', 1/mean.cp, x = data.frame(id = 1:50))

#determine the last day seen and first day not seen for each carcass so these data can be added to GenEst

surv.exp$left <- sapply(surv.exp$eventtime, function(x) search.days[which(abs(search.days - x) == min(abs(search.days - x)))] )

surv.exp$right <- sapply(surv.exp$eventtime, function(x) search.days[which(abs(search.days - x) == min(abs(search.days - x))) + 1] )

#fit these data using GenEst::cpm

cpm.fm <- cpm(~1, data = surv.exp, left = 'left', right = 'right', dist = 'exponential')

#calculate model estimated daily persistence rates

daily.cpp_est <- ppersist(pdb = cpm.fm$cell_ab$pdb_median, dist = 'exponential', t_arrive0 = rep(0, ndays + 1), t_arrive1 = rep(0.00001, ndays + 1), t_search = 0:ndays)

#simulate detection trials to estimate searcher efficiency

dets <- rbinom(100, 1, mean.se)

dets.df <- data.frame(ID = 1:length(dets), dets)

#fit searcher efficiency model using GenEst::pkm

pkm.fm <- pkm(p ~ 1, data=dets.df, obsCol = 'dets', kFixed = 0)

#calculate daily detection probabilities

daily.det <- pkm.fm$cell_pk$p_median

obs1 <- list()

obs2 <- list()

#simulate mortalities and fatality detections at the control turbines

for(j in 1:nturbines1){

for(k in 1:ndays){

#generate numbers of true carcasses

N1[i, j, k] <- y1[i, j, k] <- rpois(1, lambda = (lambda1/nhours)*nhours)

#now add the search component only a x day scale

if(k %in% search.days){

y1samp <- y1[i,j,1:k]

#for days that are searched and have a carcass, calculate the probability that it is detected and if it found

for(m in 1:k){

if(y1samp[m] > 0){

for(n in 1:y1samp[m]){

if(rbinom(1, 1, daily.det * daily.cpp[length(y1samp) - m + 1]) > 0){

obs1 <- append(obs1, data.frame(sim = i, turbine = j, day = k, y = 1, id = n, trt = 0))

y1samp[m] <- y1samp[m] - 1

}#if

}#n

}#if

}#m

y1[i,j,1:k] <- y1samp

}#if

}#k

}#j

#simulate mortalities and fatality detections at the curtailed turbines

for(j in 1:nturbines2){

for(k in 1:ndays){

#generate numbers of true carcasses

N2[i, j, k] <- y2[i, j, k] <- rpois(1, lambda = (lambda2/nhours)*nhours)

#now add the search component only a x day scale

if(k %in% search.days){

y2samp <- y2[i,j,1:k]

#for days that are searched and have a carcass, calculate the probability that it is detected and if it found

for(m in 1:k){

if(y2samp[m] > 0){

for(n in 1:y2samp[m]){

if(rbinom(1, 1, daily.det * daily.cpp[length(y2samp) - m + 1]) > 0){

obs2 <- append(obs2, data.frame(sim = i, turbine = j, day = k, y = 1, id = n, trt = 1))

y2samp[m] <- y2samp[m] - 1

}#if

}#n

}#if

}#m

y2[i,j,1:k] <- y2samp

}#if

}#k

}#j

#summarize data from the simulation to prepare for fatality calculation and account for surveys where no mortalities are found with NAs

if(length(obs1) == 0 | length(obs2) == 0){

dat.list[[i]] <- list('obs' = NA, 'dat_SS' = NA, 'dwp' = NA, 'cpm.fm' = NA, 'pkm.fm' = NA)

} else{

#combine observation data together from both surveys

obs1 <- matrix(unlist(obs1), ncol = 6, byrow = TRUE)

obs2 <- matrix(unlist(obs2), ncol = 6, byrow = TRUE)

obs <- data.frame(rbind(obs1, obs2))

colnames(obs) <- c('sim', 'turbine', 'day', 'y', 'id', 'trt')

#add other information to the observation data that GenEst needs

obs$turb_id <- paste(obs$turbine, obs$trt, sep = "_")

obs$day <- as.Date(obs$day, origin = '2019/1/1')

dat_SS <- data.frame(matrix(1, ncol = length(turbs), nrow = length(search.days)))

colnames(dat_SS) <- turbs

dat_SS$day <- as.Date(search.days, origin = '2019/1/1')

dwp <- data.frame(turb_id = turbs, dwp = 0.5)

#make a list of all the data that we need for GenEst modeling

dat.list$scenario <- scenario

dat.list$sims[[i]] <- list('obs' = obs, 'dat_SS' = dat_SS, 'dwp' = dwp, 'cpm.fm' = cpm.fm, 'pkm.fm' = pkm.fm)

}

}#i

return(dat.list)

}#fn

#genest modeling

curt.mod <- function(curt.dat){

require(GenEst)

#for each list of data that was simulated, run a GenEst model to estimate overall fatality

lapply(curt.dat$sims, function(x){

if(length(x) > 1){

eM <- estM(data_CO = x$obs, data_SS = x$dat_SS, data_DWP = x$dwp, COdate = 'day', IDcol = 'id',

model_SE = x$pkm.fm, model_CP = x$cpm.fm)

#calculate mortality for each group

Mcalc <- calcSplits(M = eM, split_CO = 'trt', data_CO = x$obs)

#gather other information on detection probability and carcass persistence

cell_pk <- x$pkm.fm$cell_pk

cell_desc <- x$cpm.fm$cell_desc

cell_ls <- x$cpm.fm$cell_ls

#move NAs through if there wasn't enough data to make a model (i.e., a treatment group had no detected mortalities)

} else{

Mcalc <- NA

cell_pk <- NA

cell_desc <- NA

cell_ls <- NA

}

#gc()

return(list('Mcalc' = Mcalc, 'se' = x$pkm.fm$cell_pk, 'cp' = x$cpm.fm$cell_desc, 'cp_param' = x$cpm.fm$cell_ls))

})

}#fn

#compilation and summary

curt.sum <- function(curt.dat, curt.mod){

require(GenEst)

#save the data and make an array to hold the model summary information

save(curt.mod, file = paste0(paste0(as.character(curt.dat$scenario), collapse = ""), '.Rdata'))

Mest <- array(NA, dim = c(curt.dat$scenario$nsim, 2, 1000))

#report a bit of summary information from the simulated data set

for(i in 1:curt.dat$scenario$nsim){

Mest[i,,] <- curt.mod[[i]]$Mcalc$M

}

print(paste('Probability of Effect Detection =', mean(apply(Mest[,1,] - Mest[,2,], 2, function(x) ifelse(quantile(x, probs = 0.025, na.rm = TRUE) < 0, 0, 1)))))

print('Overall Difference Between Treatment and Control')

print(summary(c(Mest[,1,] - Mest[,2,])))

print('Overall Ratio Between Treatment and Control')

print(summary(c(Mest[,2,]/Mest[,1,])))

#return(Mest)

}#fn

#simulate data for an example scenario using the user-defined functions above

dat1 <- curt.dat(nsim = 50000, nturbines1 = 10, nturbines2 = 10, ndays = 90,

nhours = 12, mort.i = 0.1, diff = 0.5, mean.cp = 3, mean.se = 0.5,

search.i = 3)

#then use the other functions to make GenEst models from the data then summarize the model results

mod1 <- curt.mod(dat1)

curt.sum(dat1, mod1)

#repeat as needed

#end
