# Supplementary material for: A review of the effectiveness of operational curtailment for reducing bat fatalities at terrestrial wind farms in North America
Source: PLoS One. 2021 Nov 17;16(11):e0256382. doi: 10.1371/journal.pone.0256382 (PMC8598023; doi:10.1371/journal.pone.0256382)
Supplement: S2 Appendix — (DOCX) [file pone.0256382.s004.docx]

**S2 Appendix: Meta-analysis and power analysis comparisons with additional approaches**

*Comparison of cut-in speed binning method and top meta-analysis models*

To examine the influence of bin choice for the categorical model, we examined four possible options for binning data into categories, including three options with three categories each (Options 1-3; Table A1) and an option with four categories (Option 4; not effective and not shown). We compared models using model predictions (Fig. A1), which indicated similarity among options, and we used the model log likelihoods to choose the most likely model for use in the main meta-analysis (Option 3).

Table S2-1. Comparison of binning for the meta-analysis categorical model, including Δ cut-in values in the database and number of control-treatment pairs at each Δ cut-in value (n). Options 1-3 indicate how data was binned into 3 categories (A-C) in each binning approach.

| **Δ cut-in** | **n** | **Option 1** | **Option 2** | **Option 3** |
| --- | --- | --- | --- | --- |
| 0.5 | 2 | A | A | A |
| 1.0 | 6 | A | A | A |
| 1.3 | 4 | A | A | A |
| 1.5 | 6 | A | B | B |
| 1.9 | 1 | B | B | B |
| 2.0 | 10 | B | B | B |
| 2.3 | 1 | B | C | B |
| 3.0 | 5 | C | C | C |
| 3.5 | 1 | C | C | C |
| **Model -log likelihood** | | -32.80 | -31.12 | -31.02 |


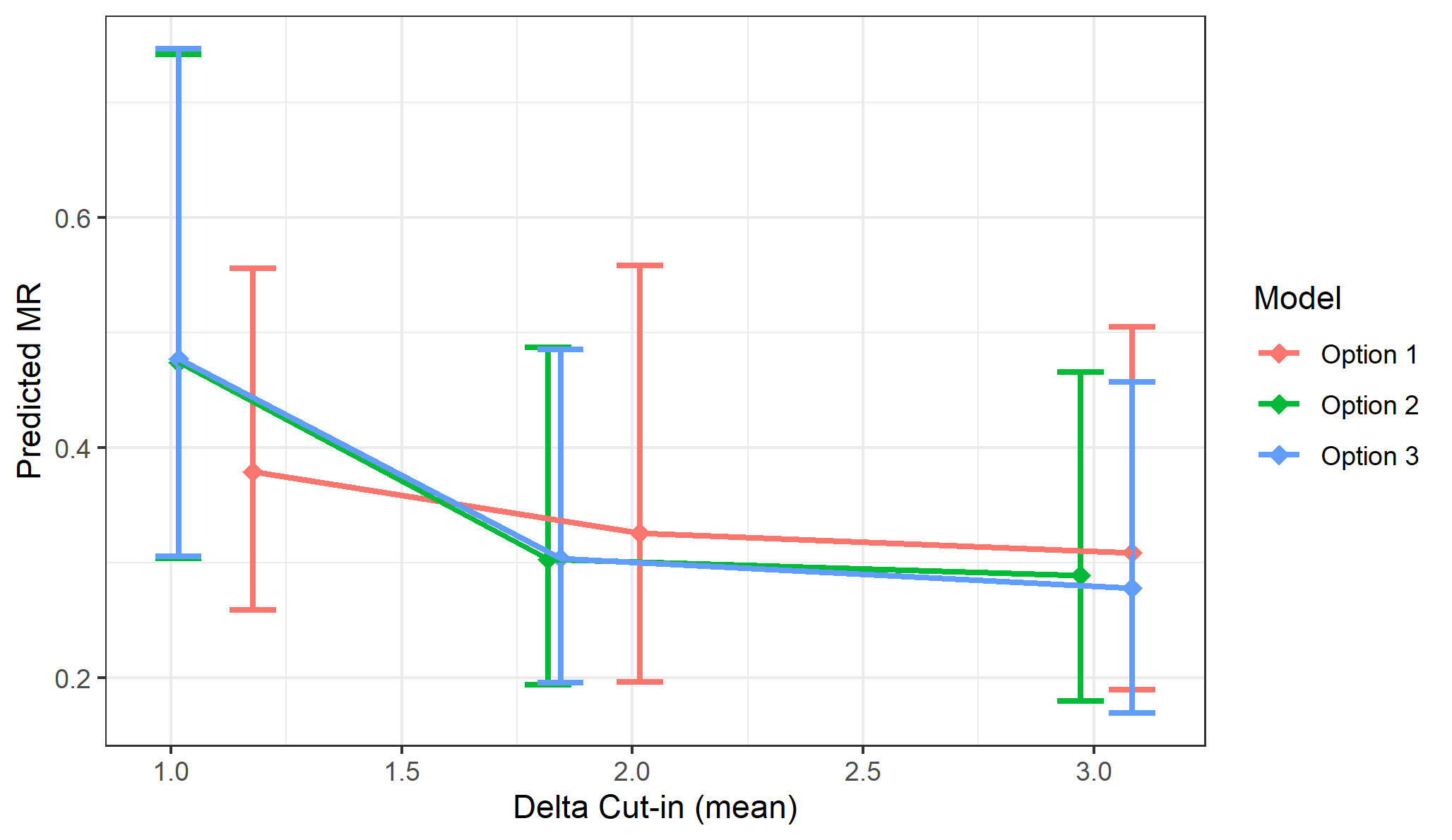


Figure S2-1. Predicted relationship between fatality ratio (MR) and Δ cut-in (delta cut-in) for the categorical model with three options for category binning (see Table 1A).

*Examination of a Potential Outlier*

To examine the potential of the Talbot Wind study as an outlier, given the funnel plot and Cook’s Distance results, we reran all models without the Talbot Wind study to examine the influence of this study on the results, including model selection (Table A2) and predictions from top models (Figure A1).

Table S2-2. Model selection results for meta-analyses of the relationship between Δ cut-in speed and bat fatalities at terrestrial wind farms in North America with the Talbot Wind study removed. Models include Δ cut-in speed (m/s) as both a continuous (cont) and categorical (cat) variable. Best fit models determined based on Akaike’s Information Criteria corrected for small sample size (AICc), with –log likelihood (-logLik) and model weight also shown.

| **Model** | **-logLik** | **AICc** | **Weight** |
| --- | --- | --- | --- |
| Δ Cut-in (cont) + Control cut-in + 1\|Site | -23.51 | 55.79 | 0.33 |
| Control cut-in + 1\|Site | -24.97 | 56.32 | 0.25 |
| Δ Cut-in (cat) + Control cut-in + 1\|Site | -22.97 | 57.28 | 0.16 |
| Δ Cut-in (cont) + RD + Control cut-in + 1\|Site | -23.12 | 57.57 | 0.14 |
| Δ Cut-in (cat) + RD + Control cut-in + 1\|Site | -22.51 | 59.08 | 0.06 |
| Δ Cut-in (cont) + Region+ Control cut-in + 1\|Site | -23.21 | 60.48 | 0.03 |
| Δ Cut-in (cat) + Region+ Control cut-in + 1\|Site | -22.39 | 61.78 | 0.02 |
| Δ Cut-in (cont) + Region + RD+ Control cut-in + 1\|Site | -22.89 | 62.78 | 0.01 |
| Δ Cut-in (cat) + Region + RD+ Control cut-in + 1\|Site | -22.03 | 64.20 | 0.00 |


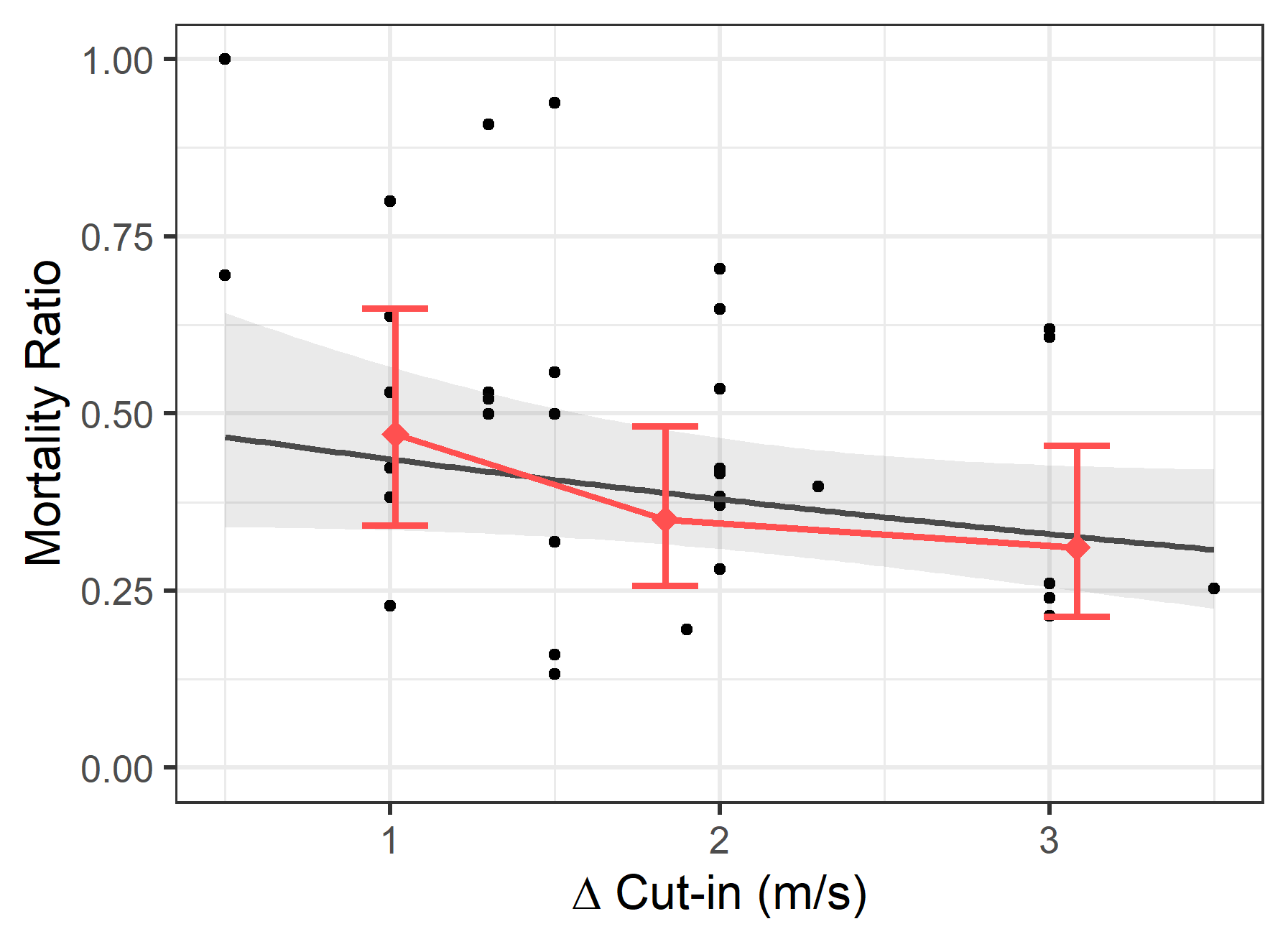


Figure S2-2. Meta-analysis estimated continuous (black line) and categorical (pink) effect of Δ cut-in speed on bat fatality ratio at North American wind energy projects with the Talbot Wind study removed. Black dots represent fatality ratios for individual studies. Categorical model points are based on mean Δ cut-in speed for the category. Error bars are 95% confidence intervals of estimates.
